# Enhanced bodily states of fear facilitates bias perception of fearful faces

**DOI:** 10.1186/s13041-020-00674-6

**Published:** 2020-11-23

**Authors:** Won-Mo Jung, Ye-Seul Lee, In-Seon Lee, Christian Wallraven, Yeonhee Ryu, Younbyoung Chae

**Affiliations:** 1 Acupuncture & Meridian Science Research Center, College of Korean Medicine, Kyung Hee University, 1 Hoegi-dong, Dongdaemun-gu, Seoul, 02447 Republic of Korea; 2 Department of Anatomy and Acupoint, College of Korean Medicine, Gachon University, Seongnam. 13306, Republic of Korea; 3 Department of Artificial Intelligence, Department of Brain Cognitive Engineering, Korea University, Seoul, 02841, Republic of Korea; 4 KM Fundamental Research Division, Korea Institute of Oriental Medicine, Daejeon, 34054, Republic of Korea

**Keywords:** Anterior cingulate cortex, Emotional face, Extrastriate body area, Fear, Interoception

## Abstract

We investigated whether enhanced interoceptive bodily states of fear would facilitate recognition of the fearful faces. Participants performed an emotional judgment task after a bodily imagery task inside a functional magnetic resonance imaging scanner. In the bodily imagery task, participants were instructed to imagine feeling the bodily sensations of two specific somatotopic patterns: a fear-associated bodily sensation (FBS) or a disgust-associated bodily sensation (DBS). They were shown faces expressing various levels of fearfulness and disgust and instructed to classify the facial expression as fear or disgust. We found a stronger bias favoring the “fearful face” under the congruent FBS condition than under the incongruent DBS condition. The brain response to fearful versus intermediate faces increased in the fronto-insular-temporal network under the FBS condition, but not the DBS condition. The fearful face elicited activity in the anterior cingulate cortex and extrastriate body area under the FBS condition relative to the DBS condition. Furthermore, functional connectivity between the anterior cingulate cortex/extrastriate body area and the fronto-insular-temporal network was modulated according to the specific bodily sensation. Our findings suggest that somatotopic patterns of bodily sensation provide informative access to the collective visceral state in the fear processing via the fronto-insular-temporal network.

## Introduction

Physiological feedback plays an important role in the perception of emotion [1], which is thought to be the subjective experience of a physiological reaction to emotional stimuli or a physiological reaction itself [2]. Debate concerning this concept has focused on whether a distinct physiological state accompanies specific emotions [3–5]. Recent studies propose that perceived emotion is a product not only of ascending emotional stimuli but also of the reciprocal interaction between the descending inference and internal states [6–9]. An accurate discrimination of facial expressions is important for social functioning. The cognitive process underlying emotional face recognition is known to be highly associated with affective disorders, such as major depressive disorder (MDD) and anxiety disorders [10]. MDD patients showed inaccurate recognition among facial expressions of six basic emotions [11] and neutral faces [12]. Surcinelli and his colleagues found more accurate recognition for fearful faces in participants with high trait anxiety than with low trait anxiety [13]. Furthermore, socially anxious individuals showed biased recognition of facial expressions to anger [14].

The core function of the brain is homeostatic regulation of the physiological state to promote survival. In contrast to the standard regulatory model in which errors are corrected via feedback, a newer model, “allostasis,” proposes a predictive regulatory model in which changes in the visceral state are anticipated and modified before they arise [15]. The concept of “predictive coding” can be explained as hierarchical Bayesian inference about the hidden causes of our sensations [16–18]. Recently, the predictive coding framework has been used in the context of interoception. “Interoceptive inference” envisions a subjective feeling (emotional feeling) as arising from predictive models of the causes of interoceptive afferents [19]. Nummenmaa et al. recently showed that different emotional states are associated with distinct bodily sensation maps, suggesting that different bodily sensation patterns originate from the different physiological conditions underlying emotion [20, 21]. Our previous study showed that somatotopic bodily sensation patterns provided a channel for inferring bodily state [22]. In this context, the somatotopical pattern of bodily sensation may provide efficient access to the collective interoceptive information. However, few studies have investigated whether manipulations of emotion-specific bodily sensation patterns can affect emotion perception.

Emerging evidence suggests that the brain functions as a generative model of the world using past experience to construct the present [23]. Interoceptive predictive coding hypothesis suggests that the conscious sense of presence depends on interactions between an interoceptive-comparator integrating ascending visceral signals [19]. Interoceptive experiences are formed from probabilistic inference about the causes of viscerosensory inputs [19]. The Embodied Predictive Interoception Coding (EPIC) model proposes that bodily predictions act as a binding pacemaker signal to create a core neuronal network workspace [24, 25]. Recently, unexpected and unconscious surges of interoceptive arousal regulated the encoding of sensory noise on perceptual awareness [26]. Insular cortex is assumed to be the principal cortical region, integrating low-level sensory prediction errors with interoceptive and attentional expectations to regulate affective salience and emotion [19, 27]. Anterior insular cortex, a center of awareness of subjective feeling, constitutes a site for multimodal integration of interoceptive and exteroceptive signals through interoceptive predictions [27]. Anterior insular cortex not only integrates bottom-up interoceptive prediction errors with top-down predictions from high-order cortical areas, but also sends descending predictions to visceral system that provide a point of reference for autonomic reflexes and for generating future awareness [28]. The bodily sensations are in part a reflection of what the brain predicts and the idea of interoceptive inference is explained within the context of body homeostasis [27]. From the perspectives of active inference framework, the emotional representation includes not only the external environment, but also interoceptive sensations from the body [27].

The present study investigated whether the synchronization with the bodily signature of fear enhance bias perception of fearful faces. We used a bodily imagery task in which participants were instructed to imagine the bodily sensations depicted on bodily sensation maps of fear or disgust. Immediately after the bodily imagery task, participants were asked to judge emotional facial expressions as fear or disgust. We hypothesized that enhanced interoceptive bodily states of fear would facilitate recognition of the fearful faces and increase activity in the fronto-insular-temporal network.

## Methods

### Participants

In total, 17 healthy student volunteers (22.8 ± 2.2 years; eight females) were recruited from Kyung Hee and Korea Universities by advertisement. No participant had a history of neurological, psychiatric, or other major medical problems, and no participants were taking medications at the time of the study. Participants were instructed not to drink alcohol or caffeine or take any medications the day before the study participation. All participants provided written informed consent before the experiments. The Institutional Review Board of Korea University approved all study protocols (KU-IRB-15–108-A-1). In the current study, we performed power analysis using Neuropower tool which provides sample size calculations for fMRI experiments [29]. A sample size of 16 participants in total was required to achieve power above 0.8 with the analysis options: cluster-forming threshold p < 0.001; alpha-level < 0.05; random-field theory correction.

### Experimental stimuli and tasks

Participants performed an emotional judgment task after a bodily imagery task inside a functional magnetic resonance imaging (fMRI) scanner. For the bodily imagery task, we generated somatotopic images, which were averaged maps of emotion-specific bodily sensations obtained in our previous study [22]. The participants of the present study were recruited from the previous study who volunteered to participate in an additional study. In the previous study, we recorded bodily sensations related to six basic emotions (happiness, sadness, fear, disgust, surprise, and anger) reported by 31 subjects on a somatotopic map. In the present study, we used fear-associated bodily sensation (FBS) and disgust-associated bodily sensation (DBS) somatotopic maps in the bodily imagery task.

**Fig. 1:**
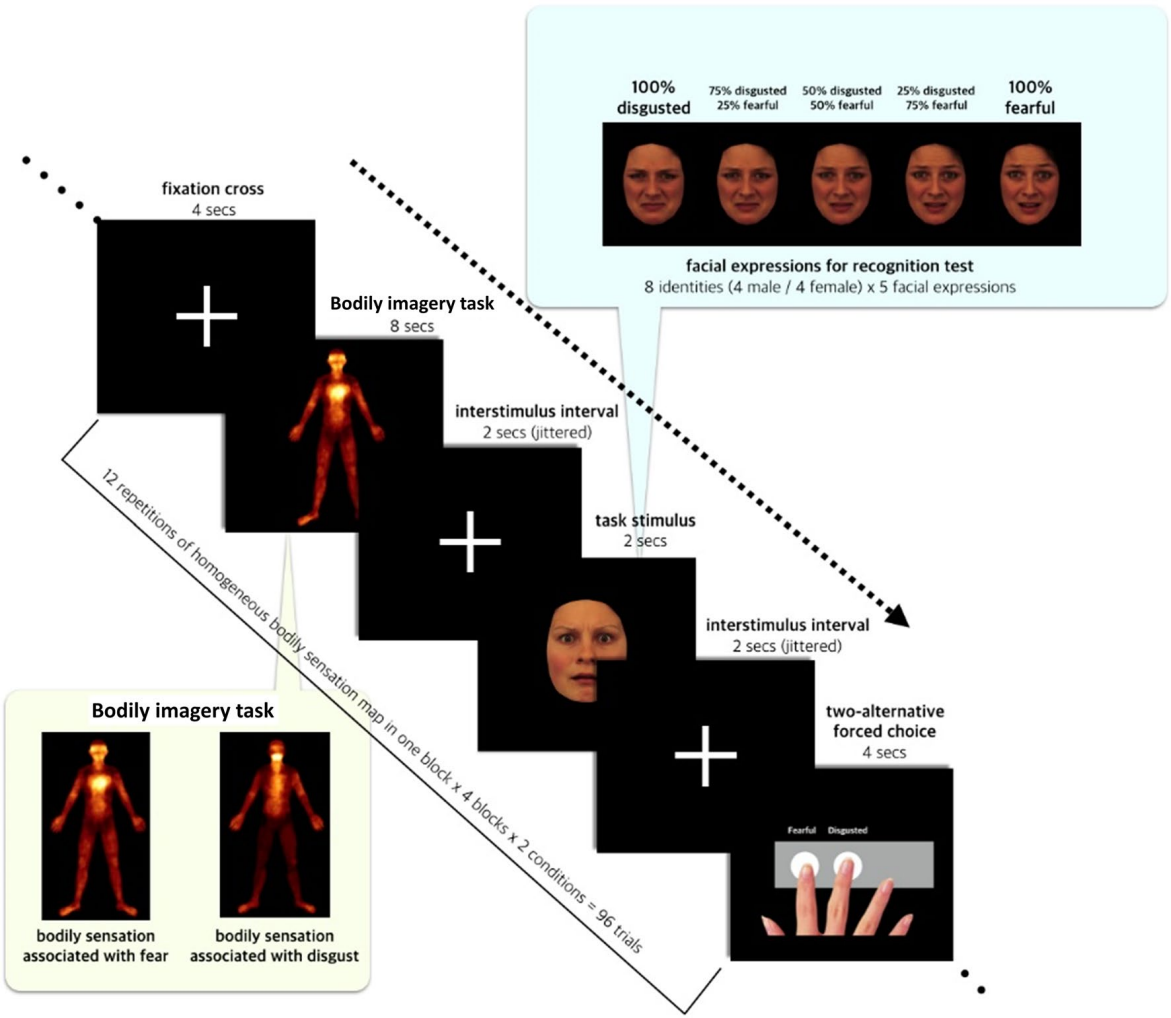
Experimental procedure during fMRI scanning. Inside the fMRI scanner, the bodily imagery task required participants to view a somatotopic map that flickered twice during an 8-s period with a 4-s cycle. Participants were instructed to imagine the bodily sensation depicted by the somatotopic pattern. The somatotopic maps were representative sensation maps for fear or disgust generated by averaging the sensation patterns recorded in subjects after watching video clips containing fearful or disgusting stimuli in our previous study. After the bodily imagery task, a face with a morphed facial expression between fear and disgust appeared for 2 s. In this two-alternative forced-choice task, participants were given 4 s to classify the expression as fearful or disgusted

During the bodily imagery task, participants were asked to imagine the bodily sensations depicted in the FBS or DBS somatotopic maps. They were told that they had to focus attention to different types of somatotopic locations and imagine feeling the bodily sensations from their own body. Importantly, subjects received no information about which emotion was associated with each somatotopic map, and the somatotopic maps were labeled as somatotopic pattern 1 and somatotopic pattern 2 throughout the procedure. The participants were given the task to guess which bodily sensation patterns were derived from the five emotions (fear, disgust, happiness, sadness, and anger). The emotional judgment task immediately followed the bodily imagery task. Participants were asked to look at an image of a facial expression, one of five morphed facial expressions ranging from fear to disgust, and then to classify the expression as fearful or disgusted (Fig. 1).

#### Generation of bodily sensation maps for fear and disgust

In our previous study, we recorded the somatotopic patterns associated with bodily sensations shortly after inducing a specific emotion [22]. After viewing an emotional video clip, participants were asked to mark the location of their bodily sensations on a somatotopic map presented on an iPad (Apple Inc., Cupertino, CA, USA) using a bodily sensation map-emotion (BSM-E) application with a template of the human body as two-dimensional frontal images. We generated a representative sensation map for each emotion (fear and disgust) by averaging the extracted FBS and DBS somatotopic patterns.

The fear-associated bodily sensations were distributed primarily over the heart and both eyes, and the disgust-associated bodily sensations were distributed mainly along a straight line connecting the mouth, neck, and chest. These patterns are in agreement with those reported by Nummenmaa et al. [20, 21]. The averaged map was visualized using an “afm hot” color map with a black-red-yellow-white color gradient, where white represented the location with the most intense sensation. We selected bodily states associated with fear and disgust, because they are primitive emotions and have opposite physiological properties [30]. Furthermore, a previous study found that a perceptual choice task successfully discriminated between fearful and disgusted faces [31] (Fig. 1).

#### Bodily imagery task

Participants viewed a somatotopic map (FBS or DBS) that flickered twice during an 8-s period with a 4-s cycle. Participants were instructed to breathe in time with the flickering of a given image. The peak of inhalation was matched to the brightest moment of the flickering. With the breathing, they were asked to imagine the strong feelings illustrated by the somatotopic pattern.

#### Morphed emotional facial expressions

Facial expressions that morphed between fear and disgust were generated for the emotional judgment task. The original stimuli were 16 pictures of emotional facial expressions (eight identities: four each of male fearful and disgusted faces and four each of female fearful and disgusted faces selected from the Karolinska Directed Emotional Faces (KDEF) database (https://www.emotionlab.se/resources/kdef)).

For each identity, five levels of facial expression from 100% fearful to 100% disgusted were generated with the morphing program, Facemorpher, using a Python library (https://pypi.python.org/pypi/facemorpher/). The five facial expressions included two original faces expressing 100% fear and 100% disgust. The remaining three faces were 75% fearful and 25% disgusted, 25% fearful and 75% disgusted, and 50% fearful and 50% disgusted.

#### Emotional judgment task

The emotional judgment task was a two-alternative forced-choice task classifying the emotional faces into fear or disgust. After the bodily imagery task, a fixation cross was displayed during an interstimulus interval; this was followed by the 2-s presentation of a face pseudo-randomly selected from the five emotional facial expressions. The duration of the interstimulus interval was pseudo-randomly selected from a range between 800 and 3200 ms, with an average of 2000 ms. After a second interstimulus interval showing the fixation cross, participants were asked to classify the presented face as fearful or disgusted. The participants were allowed 4 s to respond.

Participants were instructed to hold an fMRI-compatible four-button box in their right hand and to press the first or second button to select fear or disgust, respectively. The assignment of buttons was fixed within a session, but it was pseudo-randomly determined over the sessions. Participants were instructed to use their index and middle fingers to press the corresponding buttons. Participants were told which button was assigned to each emotion at every decision point.

### Experimental procedure

All experimental procedures were performed inside the fMRI scanner. Participants underwent a training session to familiarize them with the bodily imagery task. Before the experiments, participants were instructed to perform the bodily imagery task while viewing a continuously flickering bodily sensation map for 3 min for each emotion (fear and disgust) during the training session. The order of the fear and disgust stimuli was randomly determined.

After the training session, the main experimental session was undergone. Inside the fMRI scanner, participants performed emotional judgment tasks after a short bodily imagery task (8 s). The experiment was divided into eight sub-sessions per session. Each session had twelve trials presenting four fearful faces (two for 100% and two for 75%), four intermediate faces, and four disgusted faces (two for 100% and two for 75%), and only one type of somatotopic image (FBS or DBS) was presented in a single session. The eight sessions included four FBS images and four DBS images. The order of the eight sessions was pseudo-randomly determined with the constraint that two consecutive sessions could not use the same somatotopic information. A structural MRI scan was inserted between four-session blocks.

After the eight sessions were completed, participants were removed from the fMRI scanner and asked to evaluate the bodily sensations evoked by the bodily imagery task in terms of intensity and spatial distribution. Intensity was evaluated on a scale of 0 (no bodily sensation) to 5 (most intense bodily sensation imaginable). To determine the spatial pattern of bodily sensations under each condition, participants were asked to mark the location of their bodily sensations on a somatotopic map presented on an iPad (Apple Inc.) using a BSM-E application with a template of the human body as two-dimensional frontal images [22, 32, 33].

After being told that the bodily sensation patterns used in the bodily imagery task were derived from a previous experiment in which they had participated, the participants were asked to guess which bodily sensation patterns were derived from each emotion (fear, disgust, happiness, sadness, and anger).

### Analysis of behavioral data

The self-reported intensity levels of bodily sensations during the bodily imagery task under the FBS and DBS conditions were compared using paired *t*-tests. The spatial patterns of bodily sensation were assessed using a pixel-wise univariate *t*-test for each condition (3dttest + + , the Analysis of Functional NeuroImage (AFNI), https://afni.nimh.nih.gov/afni) within a mask of the body template. In all statistical parametric map analyses, the false discovery rate (FDR) correction was used to handle statistical inflation from multiple comparisons [22, 32, 33]. The group-level accuracy of participants’ guesses regarding the emotion used to derive the bodily sensation patterns was calculated using the F-beta score. The statistical significance of the accuracy was evaluated using a null distribution generated by 10,000 iterated random guesses among the five emotions.

Group-level emotional judgment task responses under the FBS and DBS conditions were analyzed using a 2 × 5 repeated-measures analysis of variance (ANOVA) and Tukey’s post hoc test. The statistical tests for the behavioral data were conducted using R 3.4.0. The psychometric curves were fitted using the “quickpsy” package, which uses the maximum likelihood method [34]. The group-level psychometric function was determined by plotting a cumulative Gaussian model with the respective group means of its defining parameters (i.e., threshold, slope, guessing, and lapsing rate) calculated from the individual fittings of the responses of 17 participants [35].

### Physiological arousal level measurement

During the whole fMRI scanning, heart rate was monitored using the scanner’s built-in finger-tip pulse oximeter. We compared the heart rate between FBS and DBS by examining the heart rate for 10 s including a synchronization task and the interstimulus interval after the task. Heart rate variability (HRV) derived from pulse oximetry signals was also investigated. Because 10 s time window is too narrow for the reliable HRV measurement, the inter-beat interval (IBI) was extracted from the whole procedures (4 min 30 s) of the individual sessions. IBI data were then resampled to 4 Hz using a cubic interpolation. Amplitudes of the high frequency (HF: 0.15—0.4 Hz) and low frequency (LF: 0.05—0.15 Hz) components were extracted in the time–frequency domain using python hrv library (https://github.com/rhenanbartels/hrv).

### fMRI acquisition

Structural and functional imaging was performed on a 3 T Siemens Tim Trio magnetic resonance scanner with a head coil attached. As an anatomical reference, a three-dimensional T1-weighted magnetization-prepared rapid gradient echo image dataset was obtained (TR = 2000 ms, TE = 2.37 ms, flip angle = 9°, field of view = 240 × 240 mm^2^, voxel size = 0.9 × 0.9 × 1.0 mm^3^, and 192 slices). Blood-oxygen-level-dependent (BOLD) fMRI of the whole brain was conducted using an echo planar imaging (EPI) sequence (TR = 2000 ms, TE = 30 ms, flip angle = 90°, field of view = 240 × 240 mm^2^, voxel size = 3.8 × 3.8 × 4.0 mm^3^, and 37 slices).

### fMRI analysis

Preprocessing was performed using the AFNI software package [36]. The EPI time-series data were corrected for slice timing and motion, then concatenated and transformed to a common Talairach space [37], registered to the volume with the minimum outlier fraction, spatially blurred using a 6-mm full-width-at-half-maximum (FWHM) Gaussian filter, resampled to a 3-mm isotropic resolution, and scaled to yield a mean of 100 for each voxel. Head movement during the scanning session was assessed prior to any movement correction to the fMRI data.

The nine regressors of interest represented time periods in the experimental procedure. Two regressors represented the timing of imagining bodily sensations under the FBS or DBS conditions during 8 s. Other six regressors (3 stimuli × 2 conditions) represented the timing of presentation of each level of emotional faces which are fearful (100% and 75% fearful) face, intermediate (50% fearful and 50% disgusted) face, and disgusted (100% and 75% disgusted) face under two different bodily sensation imagination conditions. The other regressor represents the timing of the emotional judgment task. These regressors of interest were fitted to the scan time course using the AFNI program, 3dDeconvolve [36]. The six motion-correction parameters of head movement assessed using the realignment procedure were entered as covariates of no interest. Regressors were convolved with a gamma variate hemodynamic response function.

Omnibus two-way within-subject ANOVA was performed to study differences in neural activation arising from 2 factors (bodily imagination and emotional face stimuli) using AFNI’s 3dANOVA3 program with the option “type” as “4”, which indicates two way repeated measure ANOVA analysis. The emotional face stimuli factor had three conditions (fearful, intermediate, and disgusted face) and the bodily imagination factor had two conditions (FBS and DBS). However, the two way ANOVA revealed no statistically significant brain activations in all main effects and interaction under family-wise error (FWE) < 0.05 (activated brain regions found with uncorrected *p* < 0.001 were described in Additional file 1: Table S1).

In the emotional judgement task, we found significant differences in the fearful face judgements from the intermediate faces between FBS and DBS conditions (63.2 ± 3.7% under FBS; 51.5 ± 4.7% under DBS). We further investigated the correlations between individual bias in behavior (the differences of fearful face judgement ratio of intermediate face between FBS and DBS) and contrasted the brain activation to the intermediate faces between FBS and DBS conditions. Brain regions showing correlations between behavioral outcomes and brain activations across individuals were tested using the analysis of covariance (ANCOVA) function provided in the 3dttest + + program (AFNI, https://afni.nimh.nih.gov/afni). However, there was no statistically significant brain activations with FWE < 0.05 (significant brain regions found with uncorrected *p* < 0.005 were described in Additional file 1: Table S2).

Secondary analysis was performed to evaluate differences in the neural response of emotional (fearful and disgusted) faces and of intermediate face under the FBS and DBS conditions individually, univariate *t*-tests were performed for two contrast images which are a contrast between fearful and intermediate face and a contrast between disgusted and intermediate face. Cluster threshold criteria were determined using Monte Carlo simulations, which resulted in a FWE-corrected significance threshold of *p* < 0.05 [38, 39]. The spatial smoothness of the data was evaluated using a modern approach, which estimates a non-Gaussian spatial autocorrelation function, greatly reducing the false positive rates (FPRs). A modified version of the AFNI 3dFWHMx software with an auto-correlation function was used to extract the actual smoothness for each participant. Then, the mean FWHM value across participants was used for the permutation test to determine the cluster-wise threshold. The permutation test was performed by generating a null distribution by randomizing the signs of the residuals among subjects. The *t*-statistic calculations were iterated 10,000 times, and the accumulated distribution of the 10,000 *t*-statistic maps were used to determine the appropriate cluster size threshold for various voxel-wise *p*-values (e.g., 0.01, 0.005, 0.001) to achieve a FPR < 0.05 [40].

Furthermore, region-of-interest analyses were performed by extracting beta estimates for each subject from a priori region of interest (ROI): the insula and the amygdala. The increased activation of amygdala and insula to social threat including facial expressions of fear and disgust are known to be robust and consistent [41–44]. We extracted ROI mask using FreeSurfer based on Deskian-Killiany atlas [45]. Beta estimates were extracted from brain responses to the fearful face and to the disgusted face for each bodily imagery task condition (FBS and DBS) separately using the 3dmaskave function in AFNI. The extracted beta estimates between bodily imagery task conditions were compared using paired *t*-tests.

A priori ROI analysis revealed statistical difference between the two bodily imagery task conditions only to the fearful face, but not to the disgusted face. The data can be shown with the raincloud plots [46]. To identify brain regions that contributed to emotional perception under each bodily sensation condition, a contrast image of the response to the fearful face after inference of the FBS (congruent emotion) and of the DBS (incongruent emotion) conditions was obtained. We used a paired *t*-test to assess the difference between FBS and DBS conditions. The same statistical thresholding method used for the neural evaluation of emotional perception was applied at the same statistical level.

A generalized form of the context-dependent psychophysiological interactions (gPPI) analysis was applied [47, 48] to the whole brain using the anterior cingulate cortex (ACC) and extrastriate body area (EBA) as seed regions. Compared with a conventional PPI analysis, the gPPI exhibits improved sensitivity and specificity [47, 48]. First, we subtracted the global trend from the original time-series data over the entire experiment. Then, the average time series was extracted for each subject in the ACC and right EBA clusters, in which group-level activity in response to the fearful face was significantly greater under the FBS than under the DBS condition. The extracted average time series of the seed regions were deconvolved using the gamma-variate hemodynamic response function as the impulse response function. The interaction PPI regressors were generated using the deconvolved seed time series by multiplying by the onset timing vectors separately for the FBS and DBS conditions. The multiplied vectors were convolved again using the gamma variate hemodynamic response function, and they were finally used in the general linear model analysis. The beta estimates associated with the PPI regressors for FBS and DBS represented the extent to which activity in each voxel correlated with activity in the ACC or EBA under each condition. The group-level analysis was applied to the beta estimates of each regressor using paired *t*-tests (FBS > DBS). We then identified voxels with significant connectivity differences under the FBS and DBS conditions using the same statistical thresholding method at the same statistical significance level (*p* < 0.05, FWE correction). All the data and code used in this study are available upon direct request as well as the conditions for its sharing or re-use.

## Results

### Bodily sensations induced by the bodily imagery task

**Fig. 2:**
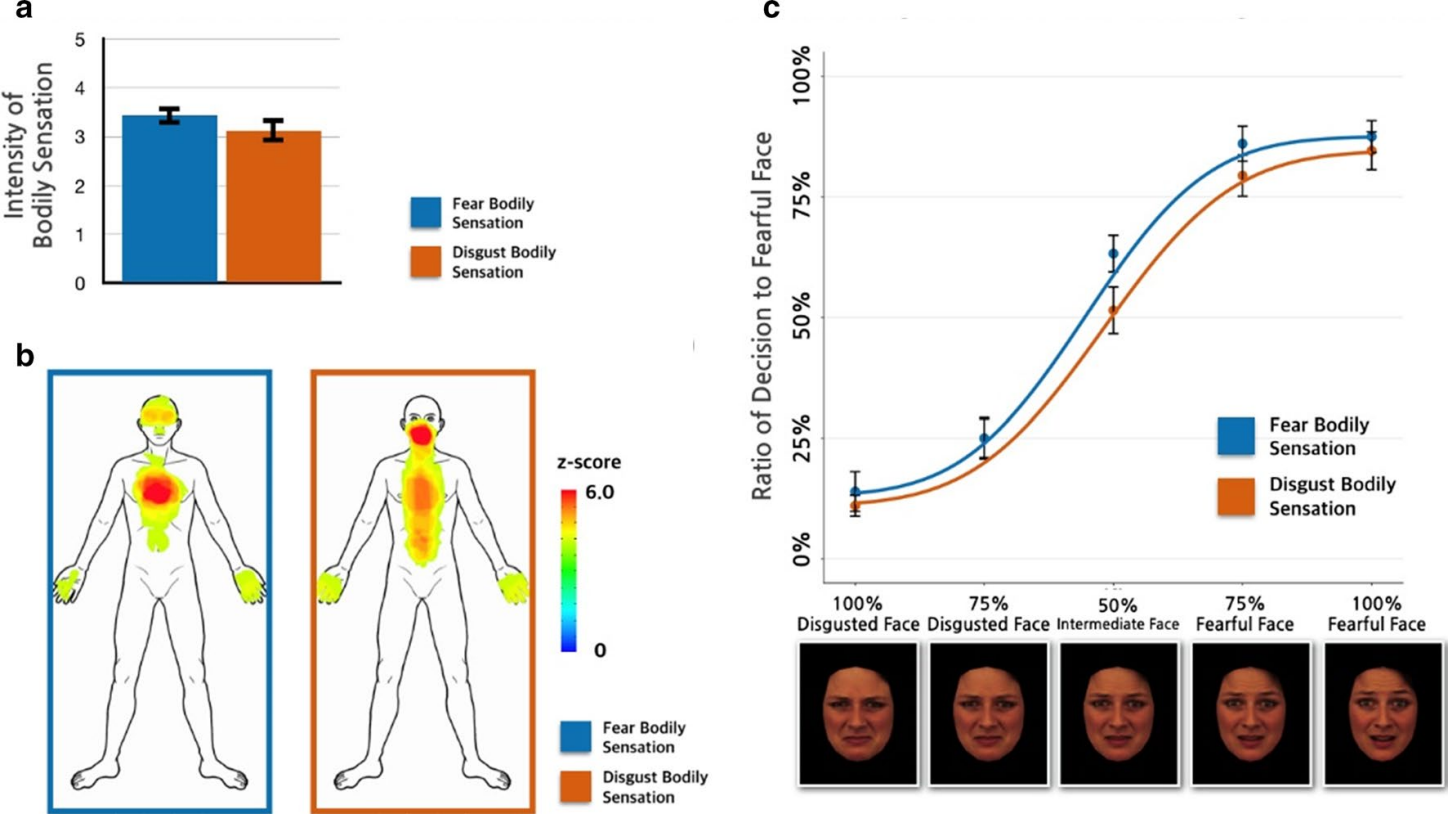
Self-reported bodily sensations according to intensity and spatial pattern and percentage of decisions favoring fearful face under congruent bodily sensation pattern. **a** Fear/disgust-associated bodily sensation (intensity). The intensity of the bodily sensations did not significantly differ between the conditions (*t* = 1.16, *p* = 0.264). The statistical parametric maps of bodily sensations induced in the bodily imagery task under each condition were visualized on a body template (FBS on the left side and DBS on the right side). **b **Fear/disgust-associated bodily sensation (spatial patterns). The location of each self-reported bodily sensation was well matched with the patterns presented in the bodily imagery task. **c** Emotional judgment task. The group-level classification ratio for each morphed face under the FBS (red) and DBS (blue) conditions. The psychometric curves fitted to the classification results are shown in the corresponding colors. The two-way repeated-measures ANOVA revealed a main effect of somatotopic pattern on the classification pattern of the emotional faces (F [1, 16] = 5.191; *p* = 0.0242). Tukey’s HSD post hoc analyses indicated that the emotional recognition bias favoring the “fearful face” was more pronounced under the FBS condition than under the DBS condition

The intensity of the bodily sensation, assessed using the 0–5 numerical rating scale, was 3.4 ± 0.2 (mean ± standard error of mean) in response to the FBS and 3.1 ± 0.3 in response to the DBS. The paired *t*-test revealed that the intensity did not significantly differ between conditions (*t* = 1.16, *p* = 0.264; Fig. 2a). The spatial patterns of the bodily sensations induced in the bodily imagery task were reported after the task was completed. The statistical parametric maps of bodily sensations induced in the bodily imagery task under each condition were visualized on a body template. The patterns of the self-reported bodily sensations were well matched with those presented in the bodily imagery task (Fig. 2b). However, the participants did not realize that their bodily sensation pattern corresponded to each emotion in the synchronized task. The group-level F-beta score for matching the emotion associated with each bodily sensation pattern was 0.146 for the FBS and 0.135 for the DBS. The F-beta scores were not significantly different from the null distribution generated by random simulation (FBS: *p* = 0.113; DBS: *p* = 0.198).

### Emotional judgment task

The group-level findings for the emotional judgment task are shown in Fig. 2c according to condition. Under the FBS condition, the percentage of prototypical disgusted faces classified as fearful was 14.0 ± 4.0%, whereas 87.5 ± 3.2% of the prototypical fearful faces were classified as fearful and 63.2 ± 3.7% of the intermediate faces (50% fearful and 50% disgusted) classified as fearful. Under the DBS condition, the percentage of prototypical disgusted faces classified as fearful was 11.0 ± 2.1%, whereas 84.6 ± 3.8% of the prototypically fearful faces were classified as fearful and 51.5 ± 4.7% of the intermediate faces (50% fearful and 50% disgusted) classified as fearful. From individual psychometric curve fittings of the responses of 17 participants, mid-points under FBS (0.59 ± 0.03%) and mid-points under DBS (0.51 ± 0.04%) were extracted. Paired *t*-test revealed that they were significantly different (*t* = 2.386, *p* = 0.029). But slope values under FBS (6.94 ± 2.28%) and slope values under DBS (12.08 ± 6.64%) were not significantly different (*t* = -0.767, *p* = 0.453).

We found a significant difference (2 × 5 repeated-measures ANOVA) in emotional judgment across somatotopic patterns (F [1, 16] = 5.191; *p* = 0.024) and across emotional facial levels (F [4, 64] = 194.7; *p* < 0.0001), but no interaction was observed (F [4, 64] = 0.901; *p* = 0.465). The post hoc test revealed that bias toward the “fearful face” in the emotional judgment task was greater under the FBS condition than under the DBS condition (*z*-score = 2.281; *p* = 0.022).

### Physiological arousal level measurement

The heart rate during bodily imagery task was 65.3 ± 1.8 in the FBS condition and 65.7 ± 1.8 in the DBS condition. The paired *t*-test revealed that the intensity did not significantly differ between the two conditions (*t* = 1.30, *p* = 0.212). The HF and LF components of HRV showed no significant effects in either condition. The normalized HF value during synchronization task was 0.42 ± 0.22 in the FBS condition and 0.39 ± 0.21 in the DBS condition. The normalized LF value during synchronization task was 0.58 ± 0.22 in the FBS condition and 0.61 ± 0.21 in the DBS condition. The paired *t*-test revealed that the normalized HRV values did not significantly differ between the two conditions (*t* = 0.538, *p* = 0.597).

### Brain responses to fearful and disgusted faces according to somatotopic condition

**Fig. 3:**
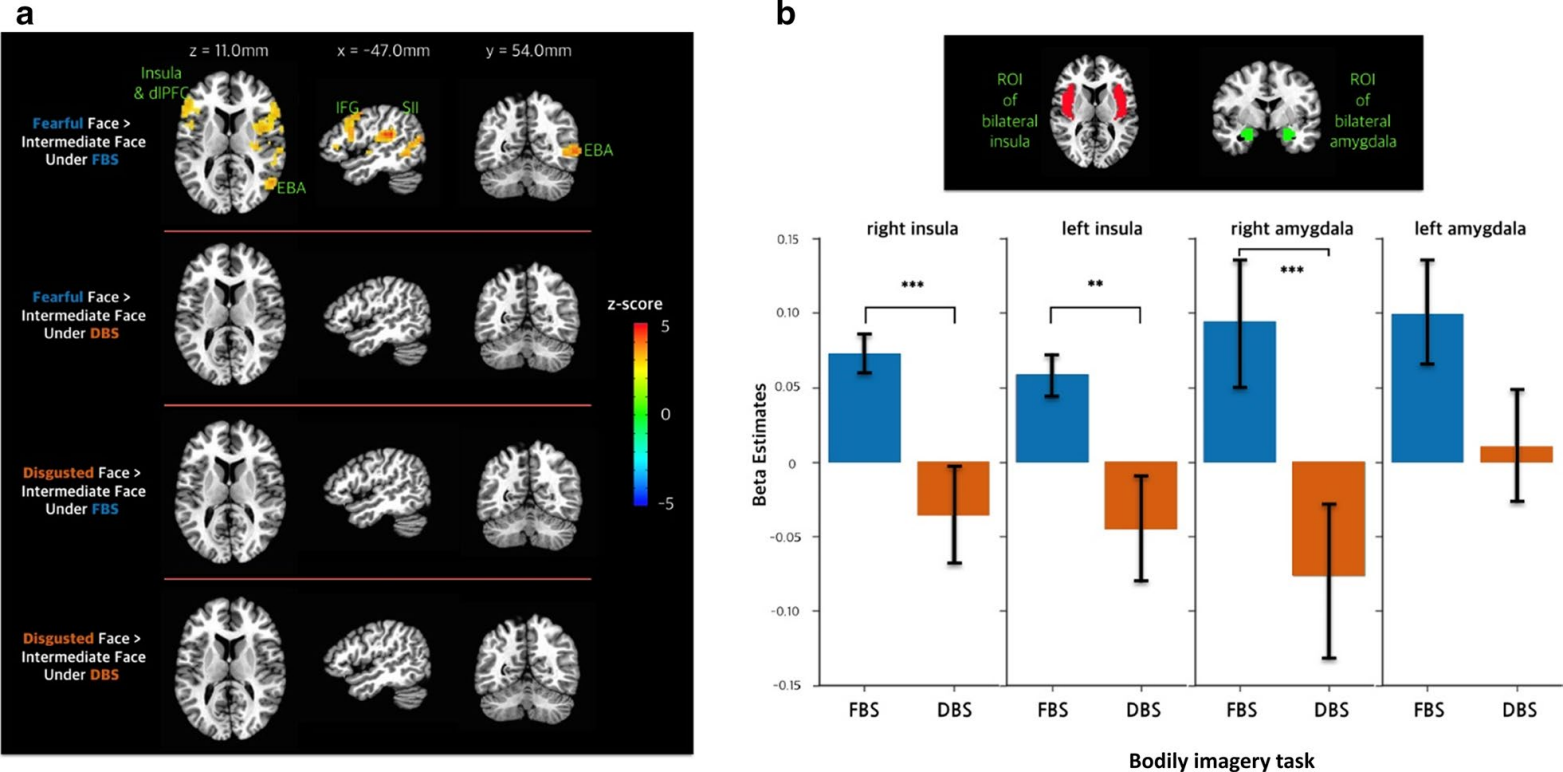
Brain responses to emotional faces according to somatotopic information (FBS and DBS). A: BOLD response to emotional face after bodily imagery task. Brain activity in response to the fearful face (fearful face > intermediate face) under the FBS condition was found in the bilateral regions of the anterior insula and dorsolateral prefrontal and inferior frontal cortices and in the right posterior insula, secondary somatosensory cortex, middle temporal gyrus (extrastriate body area), and middle occipital gyrus (*p* < 0.05; cluster-wise corrected). In contrast, the fearful face did not evoke significant brain activity under the DBS condition. Moreover, the disgusted face did not evoke a significant brain response (disgusted face > intermediate face) under the FBS or DBS condition. **a** Beta estimates for insula and amygdala (ROIs). **b** The ROI analysis of the bilateral amygdala and insula revealed that the FBS condition enhanced the brain response to fearful faces

Brain activity in response to the fearful face (fearful face > intermediate face) under the FBS (congruent) condition was found in the bilateral anterior insula, dorsolateral prefrontal (dlPFC), and inferior frontal cortices; right posterior insula; secondary somatosensory cortex; middle temporal gyrus (MTG); and middle occipital gyrus (*p* < 0.05; cluster-wise corrected; Table 1 and Fig. 3a). In contrast, the fearful face did not elicit significant brain activity under the DBS (incongruent) condition. Moreover, the disgusted face did not elicit significant brain activity under the FBS or DBS condition (disgusted face > intermediate face).

**Table 1:** Response to the fearful face versus the intermediate face under the fear-associated bodily sensation (congruent) condition

Activation	Location		Z-score	Voxels	Coordinates of the peak voxel in Talairach space (RAI)		
					x	y	z
Increased activation	Anterior insula, Posterior insula, Inferior frontal gyrus, Dorsolateral prefrontal cortex, Secondary somatosensory cortex	Right	4.6	583	− 47.2	36.0	20.5
	Middle temporal gyrus, Middle occipital gyrus	Right	4.4	277	− 36.8	71.0	17.0
	Anterior insula, Inferior frontal gyrus, Dorsolateral prefrontal cortex	Left	3.8	183	50.8	− 27.0	17.0

Subsequent priori ROI analysis of the bilateral amygdala and insula regions revealed that the brain response to the fearful face was enhanced under the FBS but not the DBS condition (Fig. 3b). We found the significant differences in three among four predefined ROIs: right insula (*t* = 3.55, *p* < 0.05; Bonferroni corrected), left insula (*t* = 3.14, *p* < 0.05; Bonferroni corrected), right amygdala (*t* = 3.45, *p* < 0.05; Bonferroni corrected). However, brain response to the disgusted face was not different between two imagination conditions. It should be noted that the activation was driven purely by the descending modulation of the prior somatotopic information derived from interoception, because the visually displayed facial images themselves were equivalent across conditions.

### Brain activity change to the fearful face between FBS and DBS

**Fig. 4:**
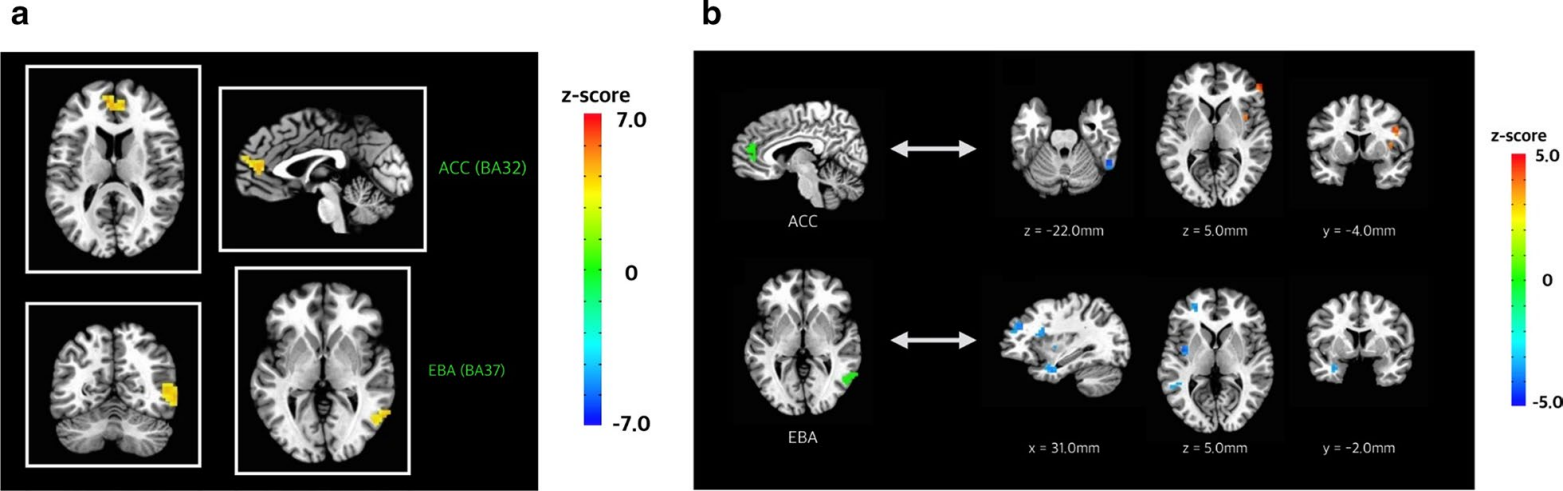
**a** Brain responses to the fearful face according to somatotopic condition. Comparison of the brain response to the fearful face under the FBS and DBS conditions revealed enhanced activity in the bilateral regions of the anterior cingulate cortex (ACC, Brodmann area 32) and the right region of middle temporal gyrus (Brodmann area 37) under the FBS condition (*p* < 0.05; cluster-wise corrected). **b** Functional connectivity of the anterior cingulate cortex (ACC) and extrastriate body area (EBA) according to somatotopic condition. The ACC and EBA, which encode different bodily sensations, were used as seed regions in the functional connectivity analysis. The dorsolateral prefrontal cortex (dlPFC), insula, operculum, fusiform gyrus, cerebellum, and extrastriate cortex (V4) showed somatotopic pattern-dependent connectivity modulation with the ACC (top). The amygdala (basolateral amygdala; BLA), insula, dlPFC, supramarginal gyrus, and left MTG showed somatotopic pattern-dependent connectivity modulation with the right EBA (*p* < 0.05; cluster-wise corrected, bottom)

There was more pronounced brain activity in response to the same fearful face under the FBS (congruent) condition than under the DBS (incongruent) condition (FBS > DBS). Enhanced activity was observed in the bilateral regions of the ACC (Brodmann area 32) and the right area of the MTG (Brodmann area 37: EBA; *p* < 0.05; cluster-wise corrected; Fig. 4a and Table 2).

**Table 2:** Comparison of responses to the fearful face under the fear-associated and disgust-associated bodily sensation conditions

Activation	Location		Z-score	Voxels	Coordinates of the peak voxel in Talairach space (RAI)		
					x	y	z
Increased activation	Anterior cingulate cortex	Right/ left	5.1	63	5.2	− 48.0	13.5
	Extrastriate body area (Medial temporal cortex)	Right	5.1	58	− 47.2	60.5	6.5

### Functional connectivity of the ACC and EBA according to somatotopic condition

Having shown that interoceptive modulation of fearful face recognition is associated with increased activity in the ACC and right EBA, we sought to determine whether dynamic changes in functional connectivity occurred in the ACC and right EBA.

The whole-brain gPPI analysis using the ACC as the seed region revealed a significant difference in bodily sensation-dependent connectivity changes between the FBS and DBS conditions in the dlPFC, insula, operculum, fusiform gyrus, cerebellum, and extrastriate cortex (V4). Compared with the DBS (incongruent) condition, the FBS (congruent) condition increased the connectivity between the ACC on the dlPFC, insula, and operculum and decreased the influence of the ACC on the fusiform gyrus, cerebellum, and V4 (Fig. 4b and Table 3).

**Table 3:** Functional connectivity of the anterior cingulate cortex according to somatotopic condition

Connectivity	Location		Z-score	Voxels	Coordinates of the peak voxel in Talairach space (RAI)		
					x	y	z
Increased connectivity	Dorsolateral prefrontal cortex	Right	5.34	17	−54.2	−41.0	6.5
	Operculum	Right	4.90	13	−40.2	−2.5	27.5
	Insula	Right	4.48	9	−36.8	−2.5	6.5
Decreased connectivity	Fusiform gyrus	Right	−4.94	23	−54.2	50	−32.0
	Cerebellum (lobule VIIIb)	Right	−4.78	9	−33.2	36	−46.0
	Occipital lobe (V4)	Left	−4.64	12	36.8	88.5	−4.0

The gPPI analysis using the right EBA as the seed region revealed a significant difference in interoceptive modulation-dependent connectivity changes in the amygdala (basolateral amygdala; BLA), insula, dlPFC, supramarginal gyrus, and left MTG. Compared with the DBS (incongruent) condition, the FBS (congruent) condition decreased the influence of the right EBA on the amygdala (BLA), insula, dlPFC, supramarginal gyrus, and left MTG (Fig. 4b and Table 4).

**Table 4:** Functional connectivity of the extrastriate body area according to somatotopic condition

Connectivity	Location		Z-score	Voxels	Coordinates of the peak voxel in Talairach space (RAI)		
					x	y	z
Decreased connectivity	Dorsolateral prefrontal cortex	Left	−6.56	81	19.2	−44.5	20.5
	Supramarginal gyrus	Left	−4.74	18	43.8	43.0	20.5
	Lingual gyrus	Left	−4.25	16	5.2	88.5	−0.5
	Insula (anterior)	Right	−4.97	16	−26.2	−23.5	17.0
	Amygdala	Left	−4.81	15	29.8	1.0	−25.0
	Middle temporal gyrus	Left	−5.00	13	43.8	43.0	3.0
	Insula (mid)	Left	−5.22	12	36.8	4.5	6.5
	Dorsolateral prefrontal cortex	Right	−4.55	11	−26.2	−44.5	24.0
	Insula (anterior)	Left	−4.44	9	33.2	−13.0	17.0

## Discussion

We found that imagining the bodily sensation patterns associated with fearful state facilitated the classification of morphed emotional faces as fearful. The neuroimaging study revealed a significant increase in the neural response to fearful faces under the FBS condition in the brain regions comprising the fronto-insular-temporal network. Subsequent analysis of the ROIs associated with fear, the amygdala and insula, revealed significantly greater activation under the FBS than under the DBS condition. Furthermore, the same fearful face elicited a more pronounced response in the ACC and EBA after the congruent bodily sensation pattern (FBS) was imagined than after the incongruent bodily sensation pattern (DBS) was. The gPPI analysis revealed that the bodily sensation pattern modulated the connectivity between the ACC and the dlPFC/mid-insula/fusiform area and between the EBA and dlPFC/anterior-insula/amygdala, which are involved in emotional processing and are components of the fronto-insular-temporal network. The ACC and EBA may modulate the processing of bodily sensation patterns related to emotion perception.

In the present study, fearful face recognition was facilitated after enhanced interoceptive bodily sates of fear. Recently, a new view of interoceptive inference has been proposed by extending the concept of predictive coding, the notion that the human brain predicts afferent signals based on a generative model, to interoception [19]. In daily life, bodily sensation patterns are constantly accompanied by emotion. Thus, we would have learned that somatotopic patterns are predictors of the emotional state in the framework of interoceptive inference. Imagining the bodily sensation of a specific emotion in the bodily imagery task increased the probability of predicting the emotional state congruent with the bodily sensation. This finding provides a concrete example of how the inference of interoceptive information can influence the process of emotional face perception. Previous findings support the influence of interoception of the visceral state on the emotional state. Previous animal studies of fear conditioning have shown that interoceptive state (hunger versus satiety) can act as a contextual factor to signal the delivery of the electric shock [49, 50]. In humans, the interoceptive state of cardiovascular arousal enhances feelings of fear and anxiety [3, 7], and cardiac signals have been shown to influence body ownership and self-identification [51–53]. Our results extend previous findings on the effect of visceral states themselves on emotion by showing that simply imagining a somatotopic pattern sensation can affect emotional processing.

Together with behavioral data, our findings provide neural evidence that the brain activation patterns arising in response to fearful faces change depending on bodily sensation. Moreover, a selective neural response to the fearful face in the amygdala and insula was observed only under the congruent bodily sensation pattern (FBS) condition. Fearful facial expressions have been shown to evoke consistent neural activity in the amygdala [54–56]. The amygdala and insula play an important role in processing the emotional content in facial expressions [54, 57, 58]. Extending the previous findings, our study suggests that bodily sensation patterns modulate emotional face perception and neural activation of the emotional perception in response to a congruent emotional state. Someone might raise the concerns in which the effect of interoceptive imagination on face recognition might be influenced by more general mechanisms, i.e., the different arousal level of fear and disgust. When we compared the heart rate and LF and HF of HRV during bodily imagery task in the current study, however, there was no significant differences of arousal level between FBS and DBS condition.

We compared the neural activity elicited by fearful faces under the FBS and DBS conditions and found that activation in the ACC and EBA was increased for the congruent bodily sensation pattern, suggesting a possible role for these brain regions as modulators of emotional processing according to bodily sensation patterns. In particular, activation of the ACC was confined to Brodmann area 32. This region corresponds to the agranular cortex, which has cortical columns with less laminar differentiation [59–61]. Although little empirical evidence for interoceptive inference has been reported, it may be that the agranular cortex in the ACC and anterior insula are involved in interoceptive prediction based on neuroanatomical characteristics [27, 60]. This notion is in agreement with evidence suggesting that the ACC plays a key role when the interoceptive state acts as a contextual factor or an unconditioned stimulus in the conditioning process [62, 63]. Furthermore, a previous study found that neuronal activation was observed in the ACC when an interoceptive threat (hyperventilation task) was anticipated by the presentation of a conditioned cue [64]. Taken together, these findings suggest that the ACC may code the prediction of the emotional state according to bodily sensation patterns.

We found that the EBA showed greater brain activation to fearful faces under interoceptive inference of congruent bodily sensation pattern than under incongruent bodily sensation pattern. The EBA is responsible for the integration of multisensory input associated with the body, including visual and tactile afferent signals [65, 66]. In addition to multisensory integration, the EBA is involved in self-body representation [67–69]. Specifically, the EBA has been shown to have more selective activity locally than in the whole body [70]. Furthermore, the EBA is involved in emotional processing and has been shown to play a key role in extracting emotional content from body expressions [71–74]. Previous studies have shown that emotional body expressions stimulated EBA activity, which was positively correlated with amygdala activity in response to emotional content [71]. Furthermore, the EBA is involved in processing associated with interoceptive signals as well as with visual information related to the body. A recent electroencephalogram study found that synchronous cardiac signals enhanced the visual processing of body, and the signal was characterized by enhanced activity in the EBA and the inferior frontal and right basal ganglia–insula regions [75]. Our finding supports the previous evidence suggesting EBA as a major channel through which interoceptive information transmits into the other brain areas. The EBA is thought to facilitate the processing of emotion associated with the somatotopic pattern of interoception information by modulating the fronto-insular-temporal network, which is involved in high-level cognitive functions [76, 77]. Analogously, our gPPI analysis revealed that the connectivity between the EBA and the vlPFC, insula, hippocampus, and amygdala, which constitute the fronto-insular-temporal network, was modulated by imagination with bodily sensation patterns. We observed significantly less connectivity between the EBA and the fronto-insular-temporal network in the congruent bodily sensation pattern than in the incongruent pattern. We regressed out the global mean of every voxel to remove correlations of no interest across the brain. However, this procedure has been shown to shift the correlation distribution to a mean near zero and force the emergence of negative correlations [78–80]. Thus, we focused on the difference in connectivity between conditions rather than on the directionality of the difference. Our findings suggest that, during the process involved in the interoceptive prediction of emotional perception, information about the specific body part associated with sensation is delivered via the EBA, as a part of the fronto-insular-temporal network, thereby modulating the processing of emotional stimuli.

Our study had several limitations. First, we used a 50% fearful 50% disgusted morphed face as a control in the evaluation of the neural response to the fearful face. Although the intermediate face contained less fearful emotional content, we cannot say that it was a neutral face. Second, interoceptive body maps of fear and disgust viewed by participants differed mainly by the involvement of face regions, i.e., eye and mouth regions. Since the bodily imagery task required them to pay attention to these body parts, their perception of the subsequent faces might be biased towards deeper processing of these face parts and thus lead to differential brain responses to fear face expression. Third, our investigation was restricted to the emotions of fear and disgust; therefore, we acknowledge that our findings are not sufficient to confirm the underlying neural pathway of bodily sensation affecting emotional processing across emotions. One possible option for control may be the neutral somatotopic maps, and future studies are needed to investigate the more generalized neural nature of this mechanism. Lastly, the sample size was quite small in the current study. Recently, many researchers raised the concerns in which relatively low power of fMRI studies contribute to a potentially highly inflated levels of false-positives [81]. The use of heuristic sample-size guidelines may give rise to increased risk of false or exaggerated results. More sufficient data are needed to ensure the reproducibility of neuroimaging research in the future. 

In summary, our behavioral and neuroimaging findings support the theory that the top-down inference of bodily sensation can facilitate the corresponding emotional perception. Somatotopic patterns of bodily sensation provide informative access to the collective visceral state. The ACC and EBA were involved in the selective modulation of bodily sensation-dependent connectivity with the fronto-insular-temporal network. Our findings suggest that perceived emotion is the product of ascending emotional stimuli and the reciprocal interaction of the descending inference about internal states.

## Supplementary information

**Additional file 1: Table S1.** Additional tables.

## Data Availability

Please contact author for data requests.

## Abbreviations

ACC: Anterior cingulate cortex
AFNI: Analysis of functional neuroimages
ANOVA: Analysis of variance
BSM-E: Bodily sensation map-emotion
DBS: Disgust-associated bodily sensation
dlPFC: Dorsolateral prefrontal
EBA: Extrastriate body area
EPIC: Embodied predictive interoception coding
FBS: Fear-associated bodily sensation
FDR: False discovery rate
fMRI: Functional magnetic resonance imaging
FPRs: False positive rates
FWE: Family-wise error
FWHM: Full-width-at-half-maximum
HRV: Heart rate variability
IBI: Inter-beat interval
KDEF: Karolinska directed emotional faces
MDD: Major depressive disorder
MTG: Middle temporal gyrus
PPI: Psychophysiological interactions
ROI: Region of interest
