# Supplementary material for: Enhanced bodily states of fear facilitates bias perception of fearful faces
Source: Mol Brain. 2020 Nov 23;13:157. doi: 10.1186/s13041-020-00674-6 (PMC7682010; doi:10.1186/s13041-020-00674-6)
Supplement: Supplementary file 1 — Additional file 1: Table S1. Additional tables. [file 13041_2020_674_MOESM1_ESM.doc]

**Additional file**

**Table S1**

Omnibus two way within-subject ANOVA was performed to study differences in neural activation arising from 2 factors (bodily imagination and emotional face stimuli) using AFNI’s 3dANOVA3 program with the option “type” as “4”, which indicates two way repeated measure ANOVA analysis. The emotional face stimuli factor had three conditions (fearful, intermediate, and disgusted face) and the bodily imagination factor had also two conditions (FBS and DBS). However, the two way ANOVA revealed no statistically significant brain activations in all main effects and interaction under FWE < 0.05 (activated brain regions found with uncorrected *p* < 0.001 were described in Supplementary Table 1).

| **Factor** | **Location** | | **F-statistics** | **Voxels (3.5x3.5x3.5mm^3^)** | **Coordinates of peak voxel in Talairach space (RAI)** | | |
| --- | --- | --- | --- | --- | --- | --- | --- |
|  |  |  |  |  | **x** | **y** | **z** |
| **Emotional Face** | **Amygdala** | right | 17.57 | 2 | 26.2 | 4.5 | -21.5 |
| **Bodily Sensation Imagination Task** | **Extrastriate Body Area** | left | 24.43 | 7 | -43.8 | 64.0 | 3.0 |
| **Interaction** | **Postcentral Gyrus** | left | 31.32 | 8 | -29.8 | 25.5 | 52.0 |


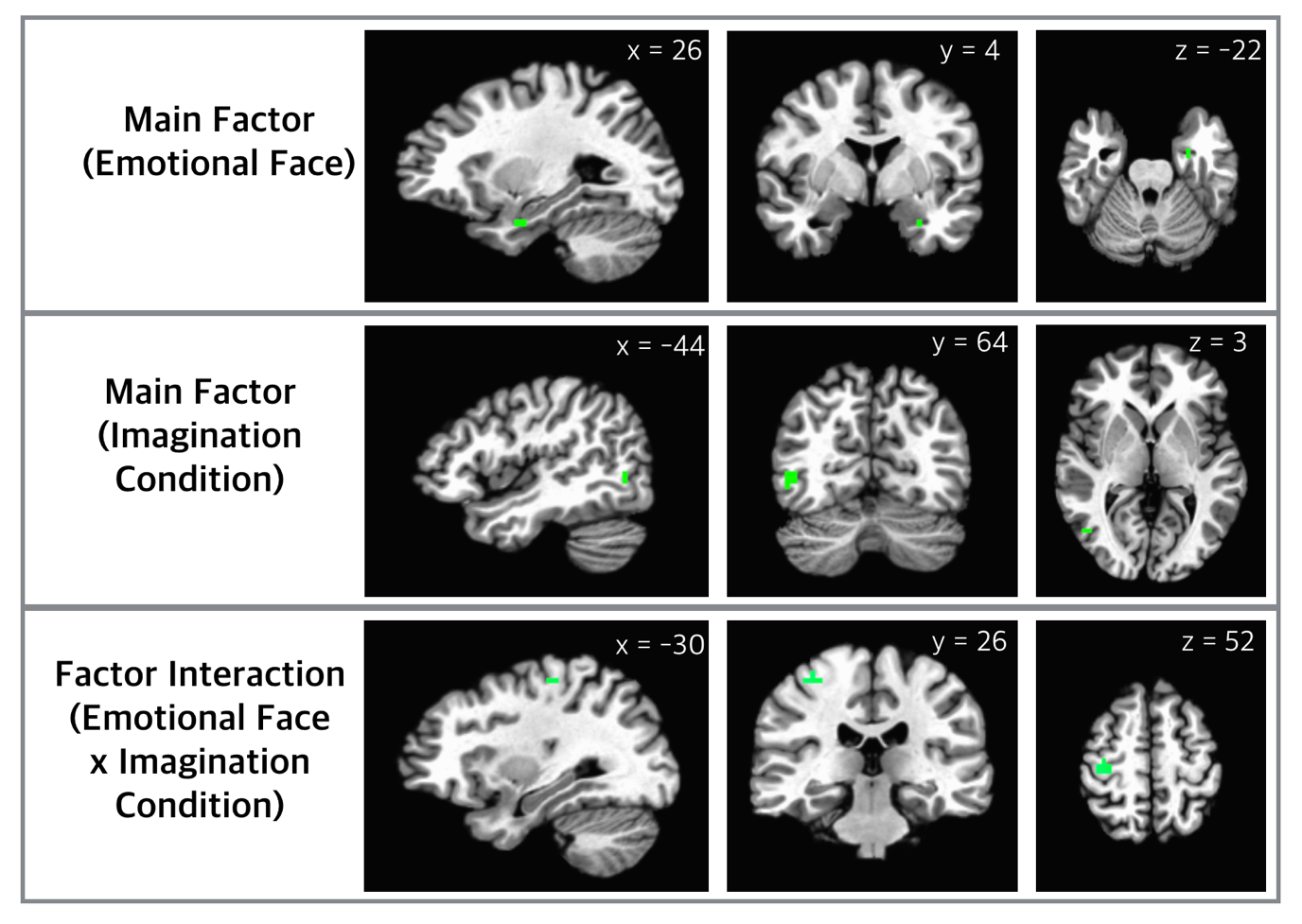


**Table S2**

In the emotional judgement task, we found significant differences in the fearful face judgements from the intermediate faces between FBS and DBS conditions (63.2 ± 3.7 % under FBS; 51.5 ± 4.7 % under DBS). We further investigated the correlations between individual bias in behavior (the differences of fearful face judgement ratio of intermediate face between FBS and DBS) and contrasted the brain activation to the intermediate faces between FBS and DBS conditions. Brain regions showing correlations between behavioral outcomes and brain activations across individuals were tested using the analysis of covariance (ANCOVA) function provided in the 3dttest++ program (AFNI, <http://afni.nimh.nih.gov/afni>). However, there was no statistically significant brain activations with family-wise error (FWE) < 0.05 (significant brain regions found with uncorrected *p* < 0.005 were described in Table S2).

| **Brain regions** | **Correlation coefficient** | ***p*-value** |
| --- | --- | --- |
| left SI | *r* = 0.734 | 0.0006 |
| cerebellum | *r* = 0.741 | 0.0005 |
| left SI | *r* = 0.741 | 0.0005 |
| right vlPFC | *r* = 0.725 | 0.0008 |
| left vmPFC | *r* = 0.718 | 0.0009 |
| right insula | *r* = 0.691 | 0.0017 |
| parahippocampus | *r* = 0.679 | 0.0021 |
| left MFG | *r* = 0.723 | 0.0008 |
